# Port catheter thickness and its correlation with complications - exploring the millimeter threshold

**DOI:** 10.1186/s13019-025-03817-9

**Published:** 2025-12-31

**Authors:** Omer Yavuz, Mehlika Iscan

**Affiliations:** Thoracic Surgery Department, Basaksehir Çam ve Sakura City Hospital, Basaksehir/Istanbul, Turkey

**Keywords:** Vascular access device, Revision surgery, Chemotherapy, Equipment design, Logistic model

## Abstract

**Background:**

Port catheters are indispensable in modern oncologic and medical care. Despite their benefits, complications such as skin erosion and related mechanical issues remain a significant concern. While numerous studies have explored prevention and management strategies, the influence of port reservoir thickness on these complications has not been systematically evaluated. This study investigates whether port thickness independently contributes to skin erosion risk, aiming to inform device selection and placement strategies.

**Patients and methods:**

This retrospective single-center study included 662 patients who underwent port catheter implantation at Istanbul Başakşehir Çam ve Sakura City Hospital between June 2022 and October 2024. Data on demographics, port brand, reservoir height, and revision status were analyzed. Appropriate nonparametric tests were used for group comparisons, and Spearman correlation and multivariate logistic regression were performed to assess the association between reservoir height and revision risk. A p-value < 0.05 was considered statistically significant.

**Results:**

Among 662 patients, 29 experienced skin erosion requiring revision, corresponding to a 4.4% overall revision rate. All revisions were due to skin erosion; no major or infectious complications occurred. Reservoir heights of the four port brands, 10.6 mm (*n* = 257), 11.6 mm (*n* = 58), 12.2 mm (*n* = 219), and 13.2 mm (*n* = 128), demonstrated a trend toward higher revision rates with increasing port thickness. Spearman correlation analysis demonstrated a weak but significant positive relationship between reservoir height and revision risk (*r* = 0.168, *p* < 0.001), indicating a size-dependent trend. In multivariate logistic regression, both port reservoir height and anti-VEGF therapy emerged as independent predictors of revision. Compared with 10.6-mm ports, 12.2-mm and 13.2-mm ports were associated with 4.47- and 10.39-fold higher revision risks (*p* = 0.023 and *p* < 0.001, respectively). Anti-VEGF therapy increased the revision risk by approximately 2.9-fold (*p* = 0.022).

**Conclusions:**

Port reservoir thickness is an independent predictor of skin erosion requiring revision. Thicker ports were linked to higher complication rates, particularly in patients receiving anti-VEGF therapy, underscoring the importance of device selection and design considerations in clinical practice. These findings suggest that thinner-profile ports should be preferred when feasible. Prospective studies are needed to validate these findings and establish evidence-based recommendations for port selection and placement.

## Introduction

Since their introduction in the 1980 s, port catheters have become widely used, offering rapid, safe, and stable venous access [1]. Their ease of use and high patient compliance have made them an essential component of treatment, particularly for cancer patients.

Although port catheters have been widely used in clinical practice for many years, the risk of various complications remains a significant concern. Complications such as skin erosion can adversely affect patient comfort and the treatment process, often necessitating additional surgical interventions. Thicker reservoirs may exert greater cutaneous pressure, potentially compromising local perfusion and predisposing to erosion. Skin erosion rates have been reported to range from 0.6% to 10% in various studies, and this complication likely develops over the port septum due to repetitive access at the same puncture site of the port catheter [2]. To prevent such complications, it is essential to consider factors like port reservoir thickness (height) in port catheter design. However, there is a lack of sufficient studies in literature addressing this issue. Therefore, we aimed to investigate the impact of port catheter reservoir height on complications and revision rates.

Depending on the insertion site (subclavian, internal jugular, or femoral), complications may vary, including pneumothorax, severe bleeding, hematoma, arrhythmia, and thrombosis [3].

Beyond insertion-related issues, late pocket-site complications such as skin erosion have also been reported. Although relatively uncommon, previous studies have primarily focused on wound dehiscence or drug-related causes, particularly anti-VEGF therapy, rather than on mechanical or device-related factors. Recent experimental and computational analyses have shown that specific port design features, including chamber geometry and edge curvature, can affect internal flow patterns and wall shear stress, thereby influencing the likelihood of mechanical complications [4]. Complementary imaging-based studies have further revealed that microstructural irregularities and wall heterogeneity can occur in both silicone and polyurethane catheters, suggesting that material composition and manufacturing precision may also contribute to long-term mechanical instability [5]. Additionally, plastic-based ports have been experimentally shown to develop microdeformations on the reservoir floor after repeated punctures, leading to altered flow dynamics, reduced flushing efficacy, and potential biofilm accumulation [6]. Meanwhile, wound-healing impairments associated with anti-VEGF agents have been documented in broader surgical and dermatologic contexts as well [7–9].

Skin erosion, although relatively uncommon, remains a clinically important late complication of port catheters. Reported incidences range between 0.6% and 10%, depending on patient population and technique [2, 10–12]. Burris and Weis demonstrated that the incidence of erosion could be reduced from 3.2% to < 1% by increasing implantation depth (0.5–2 cm) and avoiding high-profile reservoir designs [13]. Collectively, these findings suggest that inadequate subcutaneous coverage, excessive reservoir height, and repetitive puncture at the same septal site may act as mechanical contributors to erosion-parameters that have not been systematically analyzed in prior studies.

However, characteristics such as the port reservoir’s thickness (height) or profile may also influence tissue pressure and erosion risk, a relationship that has not been adequately addressed in the literature.

While numerous studies have focused on preventing and managing common complications associated with port catheters, limited attention has been given to skin erosion despite its potential impact on patient comfort and treatment continuity. As emphasized by Thiel et al., adherence to standardized protocols during port placement and maintenance can substantially reduce complication rates [14]. However, no standardized approach currently exists for the prevention or revision of skin erosion, which remains an underrecognized complication. In this context, identifying device-related factors such as port reservoir height and patient- or treatment-related factors, including anti-VEGF therapy, may contribute to developing evidence-based strategies to minimize erosion risk and guide revision management.

The primary aim of this study is to evaluate the impact of port catheter reservoir height on the incidence of skin erosion. In addition, this study seeks to identify key risk factors, including both device-related parameters and treatment-related factors such as anti-VEGF therapy, that may contribute to this complication. By addressing these factors, we aim to support the development of standardized, evidence-based strategies for managing cases requiring revision and to provide a framework for future clinical and research efforts.

## Materials and methods

### Study design and patient selection

This retrospective study included patients who underwent port catheter placement due to a cancer diagnosis at the Thoracic Surgery Department of Istanbul Başakşehir Çam ve Sakura City Hospital between June 2022 and October 2024. All procedures were performed by a single surgeon.

The inclusion criteria were: (1) age over 18 years, (2) port catheter placement for oncological treatment, and (3) absence of pregnancy. Patients were excluded if: (1) they were under 18 years old, (2) the port catheter placement was unsuccessful, or (3) they were pregnant.

Patient data were obtained from the hospital information system, ensuring adherence to ethical and privacy standards. Data collected included patient age, gender, primary diagnosis, date of port catheter placement, side and vein of placement, and whether revision was required for skin erosion. The port brands and their respective reservoir heights (thicknesses) were also recorded. Due to legal considerations, port brands were anonymized as A, B, C, and D. All four port catheter brands were oval-shaped, 8-F sized, single-lumen, and made of a silicone/polyurethane hybrid; however, their reservoir heights varied. These variations in heights were analyzed as a potential risk factor for skin erosion. The selection of port brand was dictated solely by hospital inventory availability at the time of implantation, independent of medical criteria, patient characteristics, or surgeon preference.

### Surgical technique

All procedures were performed in a day-surgery setting under local anesthesia, maintaining proper sterilization. Imaging guidance was not used as per institutional protocol and the low-resource nature of the clinical setting.

Preoperatively, all patients underwent routine evaluations, including complete blood count, coagulation parameters, serology, and imaging studies. The port placement followed a standardized approach:

1. Using anatomical landmarks, the jugular or subclavian vein was punctured, guided by the sternocleidomastoid muscle or the lateral one-third of the clavicle.
2. A guidewire was advanced using the Seldinger technique, followed by appropriate dilation.
3. The permanent catheter was inserted into the selected vein.
4. A subcutaneous pocket was created through an approximately 3 cm incision, located 3–4 cm below the midclavicular line.
5. A tunnel was formed between the venipuncture site and the reservoir pocket, allowing the catheter to be routed into the pocket.
6. The catheter was connected to the reservoir, tested with saline, and secured.
7. The skin was closed with absorbable sutures, ensuring that the reservoir pocket extended inferiorly, keeping the port needle entry point distant from the incision during use.
8. A standard posteroanterior chest X-ray was performed to verify catheter positioning and rule out complications.

The preference for right-sided internal jugular access was consistent with prior literature emphasizing lower thrombotic risk compared with left-sided approaches [15–17].

### Port revision protocol

For patients who developed skin erosion, a standardized revision protocol was applied:

1. Clinical Evaluation: Vital signs were monitored, infection markers (such as leukocytosis and acute-phase reactants) were assessed, and any discharge at the erosion site was noted.
2. Initial Management: If infection markers were elevated, antibiotic therapy was initiated. A Betadine-based dressing was applied regardless of discharge presence.
3. Port Culture: If discharge was present, a bacterial culture was obtained. If growth was detected, the port catheter was removed to prevent bacteremia, and these patients were excluded from further analysis.
4. Surgical Revision:

- If salvageable, the eroded skin area was widened, the port was mechanically cleaned, and the wound edges were debrided.
- The reservoir was repositioned in the debrided pocket and secured.
- If the skin overlying the port had significantly thinned along its entire length (subjectively perceived as the port being “too large” for the patient), the entire port system, including the reservoir and catheter, was replaced.

5. Post-Revision Care: Patients who underwent revision were discharged the same day after a control chest X-ray, similar to those undergoing primary port catheter implantation.

**Fig. 1:**
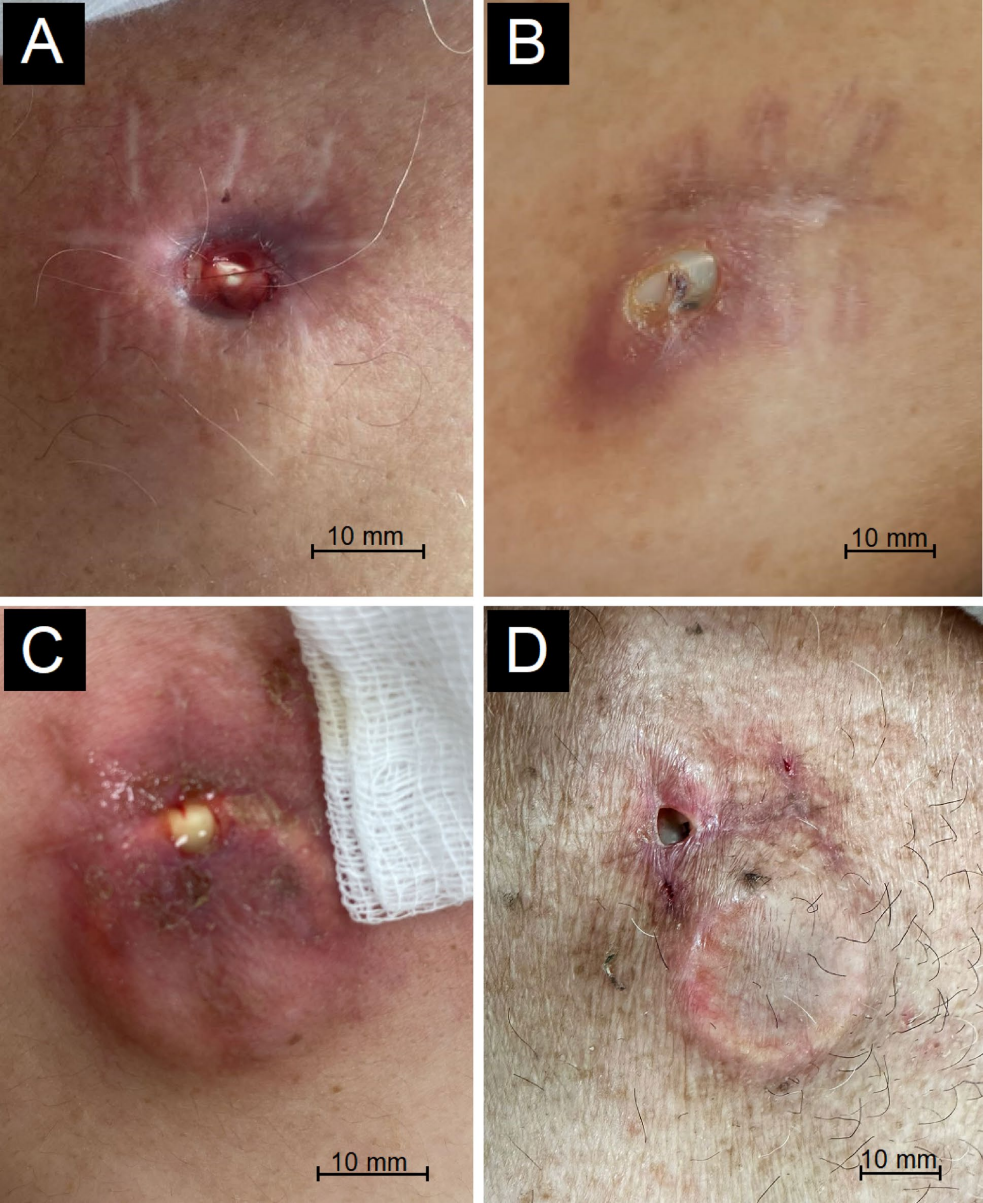
Representative clinical photographs showing skin erosion over the implanted port site in different patients prior to revision surgery. (**A**) 64-year-old male with gastric cancer, right internal jugular port; revision at day 151. (**B**) 70-year-old female with pancreatic cancer, right subclavian port; revision at day 245. (**C**) 60-year-old female with colon cancer, right internal jugular port; revision at day 76. (**D**) 60-year-old male with rectal cancer, right internal jugular port; revision at day 173. Each image demonstrates pre-revision cutaneous breakdown with exposure of the port reservoir or septum, occurring at varying degrees of severity. (Scale bars: 10 mm)

**Fig. 2:**
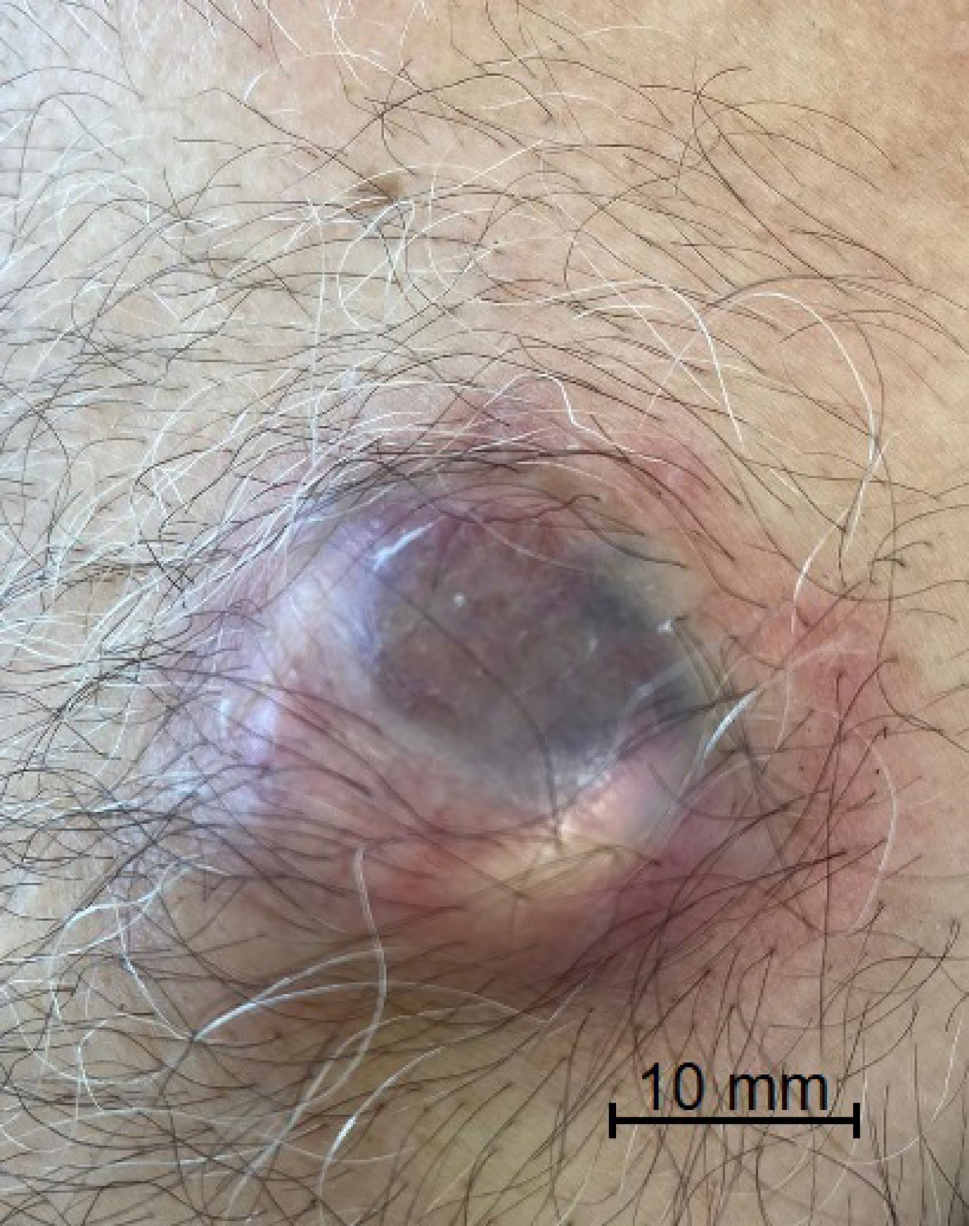
Clinical photograph showing marked thinning of the skin over the port reservoir, with the underlying silicone septum contour discernible through the attenuated dermis; 69-year-old male with colon cancer, right internal jugular port, erosion developed approximately 8 months after implantation. This condition represents a pre-erosive stage preceding full skin breakdown and necessitated port revision to prevent impending exposure. (Scale bar: 10 mm)

Examples of skin erosion observed in patients are shown in Fig. 1 and port catheters undergoing revision due to significant skin erosion are illustrated in Fig. 2.

### Statistical analysis

Normality was assessed using the Shapiro–Wilk test. Descriptive analyses were performed to evaluate clinical and demographic characteristics. Statistical comparisons were conducted using Chi-square, Fisher’s Exact, ANOVA, or Kruskal–Wallis tests, depending on variable type and distribution. Correlation between continuous variables was examined using Pearson (parametric) and Spearman (non-parametric) correlation tests.

Multivariate logistic regression analysis was performed to assess independent predictors of revision. Variables included in the model were age, sex, body mass index (BMI), smoking status, comorbidities, oncologic treatment type (including anti-VEGF therapy), port reservoir height, chronic steroid use, organ failure (e.g. hepatic, renal, or cardiac insufficiency), and thyroid dysfunction.

All statistical analyses were conducted using SPSS Statistics version 22 (IBM Corp., Armonk, NY, USA), and a p-value < 0.05 was considered statistically significant.

All port catheters were inserted by the same experienced thoracic surgeon using a standardized landmark-based technique, as imaging guidance was not available in the institutional protocol. Although ultrasound-assisted cannulation may improve venous puncture accuracy, it is unlikely to influence long-term skin erosion risk, which primarily depends on subcutaneous tissue tension and device–skin interaction rather than puncture-site precision. Procedural consistency was thus maintained across all cases, minimizing operator-related variability.

### Ethical approval

Ethical approval for this retrospective study was obtained from the Başakşehir Çam ve Sakura City Hospital Ethics Committee (Decision No: 2024-39, Date: 11.12.2024).

## Results

A total of 662 consecutive port catheter placements were performed by a single surgeon at a single center. Skin erosion requiring revision occurred in 29 patients (4.4%). Patient data were retrospectively retrieved from the hospital information system, and unsuccessful placements were excluded from the analysis.

Of the 662 patients, 400 (60.4%) were male and 262 (39.6%) were female. Port catheters were predominantly placed on the right side (96.2%), and the internal jugular vein was used in 74.8% of cases (Table 1).

**Table 1:** Baseline demographic and clinical characteristics of the study population

Variable	*n* (%) or Median (Range, IQR)
Total Patients (n)	662
Age (years)	62, (18–93), IQR: 16
Sex	
Male	400 (60.4%)
Female	262 (39.6%)
Height (cm)	167, (135–194), IQR: 12
Weight (kg)	71, (39–113), IQR: 18
BMI (kg/m²)	25.4, (15.4–50.2), IQR: 6
Placement Side	
Right	637 (96.2%)
Left	25 (3.8%)
Access Vein	
Jugular	495 (74.8%)
Subclavian	167 (25.2%)
Smoking	181 (27.3%)
Chronic Steroid Use	11 (1.7%)
Comorbidities	
Diabetes Mellitus	186 (27.8%)
Hypertension	249 (37.6%)
Thyroid Dysfunction	44 (6.6%)
Hypothyroidism	41 (6.2%)
Hyperthyroidism	3 (0.4%)
Organ Failure	33 (5%)
Renal	11 (1.6%)
Hepatic	4 (0.6%)
Cardiac	18 (2.8%)
Oncologic Treatment	
Fluoropyrimidine	551 (83.2%)
Platinum-based agents	515 (77.8%)
Topoisomerase inhibitors	132 (19.9%)
Microtubule inhibitors	105 (15.9%)
Antimetabolites	9 (1.4%)
Alkylating agents	14 (2.1%)
Anti-VEGF agents	63 (9.5%)
Anti-EGFR agents	34 (5.1%)
Anti-angiogenic agents	2 (0.3%)
HER2-targeted agents	8 (1.2%)
Immunotherapy	4 (0.6%)

As shown in Table 1, fluoropyrimidine- and platinum-based regimens were the most commonly administered chemotherapeutic agents via the port catheter. Targeted agents, particularly anti-VEGF therapies, were used in a smaller subset of patients (9.5%) (Table 2).

**Table 2:** Baseline characteristics according to Port reservoir height

Variable	10.6 mm (*n* = 257)	11.6 mm (*n* = 58)	12.2 mm (*n* = 219)	13.2 mm (*n* = 128)	*p*-value
Age (years)	62.0 [53.0–70.0]	62.5 [50.5–70.0]	62.0 [55.0–69.0]	61.0 [54.0–68.0]	0.732
BMI (kg/m²)	25.3 [22.6–28.0]	24.3 [22.3–29.9]	25.5 [23.1–29.0]	25.8 [23.5–29.3]	0.420
Male sex	154 (59.9%)	37 (63.8%)	132 (60.3%)	77 (60.2%)	0.958
Diabetes mellitus	67 (26.1%)	12 (20.7%)	66 (30.1%)	39 (30.5%)	0.408
Hypertension	96 (37.4%)	21 (36.2%)	82 (37.4%)	50 (39.1%)	0.982
Thyroid dysfunction	15 (5.8%)	3 (5.2%)	16 (7.3%)	10 (7.8%)	0.823
Any organ failure	16 (6.2%)	3 (5.2%)	9 (4.1%)	5 (3.9%)	0.680
Smoking	69 (26.8%)	13 (22.4%)	62 (28.3%)	37 (28.9%)	0.801
Chronic steroid use	1 (0.4%)	0 (0%)	7 (3.2%)	3 (2.3%)	0.530
Anti-VEGF therapy	22 (8.6%)	3 (5.2%)	22 (10.0%)	16 (12.5%)	0.401
Fluoropyrimidines	213 (82.9%)	50 (86.2%)	182 (83.1%)	106 (82.8%)	0.938
Platinum agents	202 (78.6%)	46 (79.3%)	172 (78.5%)	95 (74.2%)	0.755
Topoisomerase inhibitors	52 (20.2%)	10 (17.2%)	43 (19.6%)	27 (21.1%)	0.941
Microtubule inhibitors	44 (17.1%)	8 (13.8%)	30 (13.7%)	23 (18.0%)	0.640

Demographic, clinical, and treatment-related variables were compared among patients grouped by port reservoir height (10.6 mm, 11.6 mm, 12.2 mm, and 13.2 mm). Age, BMI, sex distribution, comorbidities (diabetes, hypertension, thyroid dysfunction, organ failure), smoking and steroid use, as well as major chemotherapy regimens (fluoropyrimidines, platinum agents, topoisomerase and microtubule inhibitors, anti-VEGF therapy) were analyzed. No statistically significant inter-group differences were observed, confirming comparable baseline profiles across all port reservoir height categories (Table 3).

**Table 3:** Distribution of chemotherapy regimens according to Port reservoir height

Port Brand	A	B	C	D
**Port reservoir height (mm)**	**10**,**6**	**11**,**6**	**12**,**2**	**13**,**2**
n	257	58	219	128
Fluoropyrimidine	213 (82.9%)	50 (86.2%)	182 (83.1%)	106 (82.8%)
Platinum-based agents	202 (78.6%)	46 (79.3%)	172 (78.5%)	95 (74.2%)
Topoisomerase inhibitors	52 (20.2%)	10 (17.2%)	43 (19.6%)	27 (21.1%)
Microtubule inhibitors	44 (17.1%)	8 (13.8%)	30 (13.7%)	23 (18.0%)
Antimetabolites	3 (1.2%)	2 (3.4%)	2 (0.9%)	2 (1.6%)
Alkylating agents	5 (1.9%)	3 (5.2%)	6 (2.7%)	0 (0.0%)
Anti-VEGF agents	22 (8.6%)	3 (5.2%)	22 (10.0%)	16 (12.5%)
Anti-EGFR agents	13 (5.1%)	5 (8.6%)	11 (5.0%)	5 (3.9%)
Anti-angiogenic agents	0 (0.0%)	1 (1.7%)	1 (0.5%)	0 (0.0%)
HER2-targeted therapy	2 (0.8%)	1 (1.7%)	3 (1.4%)	2 (1.6%)
Immunotherapy	1 (0.4%)	1 (1.7%)	1 (0.5%)	1 (0.8%)

The table summarizes the distribution of chemotherapy regimens administered through ports with different reservoir heights (10.6 mm, 11.6 mm, 12.2 mm, and 13.2 mm). Major cytotoxic and targeted agent classes, including fluoropyrimidines, platinum compounds, topoisomerase and microtubule inhibitors, anti-VEGF and anti-EGFR agents, anti-angiogenic drugs, HER2-targeted therapies, and immunotherapies, are presented as number (%) of patients treated within each group. The overall distribution of treatment categories was comparable among height groups, indicating no significant imbalance in chemotherapy exposure across device types.

### Port reservoir height and revision risk

A total of 662 patients received one of four different brands of ports (A, B, C, and D), depending on hospital inventory availability at the time of implantation. All four models were oval-shaped, single-lumen ports with an 8 French catheter size and a silicone/polyurethane hybrid composition. However, their reservoir heights varied among brands, ranging from 10.6 mm to 13.2 mm.

While the overall design of the four port brands was comparable, subtle differences were identified in both structural and material characteristics. Variations in reservoir height, base composition, width, diameter, and weight were systematically analyzed to determine their potential association with skin erosion and revision risk as discussed later in the section on Structural and Material Characteristics of the Port Systems.

Port reservoir heights, listed from thinnest to thickest, were as follows:

- Brand A: 10.6 mm.
- Brand B: 11.6 mm.
- Brand C: 12.2 mm.
- Brand D: 13.2 mm.

Revision rates varied across port brands, demonstrating a clear trend toward higher revision frequency with increasing reservoir height (Table 4). Follow-up duration was comparable among groups, with a median of 313.5 days for the overall cohort.

**Table 4:** Characteristics of Port catheters by brand

Revision cases	
No revision required	633 (95.6%)
Revision required	29 (4.4%)

Port reservoir height distribution by brand	
Brand A (10.6 mm)	257 (38.8%)
Brand B (11.6 mm)	58 (8.7%)
Brand C (12.2 mm)	219 (33.1%)
Brand D (13.2 mm)	128 (19.3%)

Revision cases by port reservoir height and brand	
Brand A (10.6 mm)	3 (10.3% of revisions)
Brand B (11.6 mm)	1 (3.4% of revisions)
Brand C (12.2 mm)	11 (37.9% of revisions)
Brand D (13.2 mm)	14 (48.3% of revisions)

Follow-up period in days (median)	
Brand A (10.6 mm)	248.9 (87)
Brand B (11.6 mm)	288.01 (294)
Brand C (12.2 mm)	462.4 (407)
Brand D (13.2 mm)	200.6 (210)
All patients (*n* = 662)	313.5 (273)

The mean follow-up duration for all patients was 313.5 days (median: 273 days). Among revision cases, the mean interval from implantation to revision was 120 ± 70 days (median: 97 days; range: 21–302 days). Revisions occurred as early as day 21 and as late as day 302 after port implantation, with most cases clustering within the first six months.

Spearman correlation analysis demonstrated a weak yet statistically significant positive association between port reservoir height and revision rates (*r* = 0.168, *p* < 0.001; Table 5). Comparison of revision frequencies across height groups also revealed a significant difference (*p* < 0.001), indicating a trend toward higher revision risk with increasing reservoir height.

**Table 5:** Spearman’s rank correlation between Port reservoir height and revision risk

Variables	Spearman’s *r*	*p*-value
Port reservoir height vs. revision rate	0.168	< 0.001

Logistic regression analysis revealed that greater port reservoir height was independently associated with an increased likelihood of revision. Compared with 10.6 mm ports, 12.2 mm ports exhibited a 4.47-fold higher revision risk (*p* = 0.023, 95% CI: 1.22–16.26), while 13.2 mm ports showed a 10.39-fold higher risk (*p* < 0.001, 95% CI: 2.93–36.89). The risk increased markedly once reservoir height exceeded 12 mm. Full statistical results are summarized in Table 6.

**Table 6:** Logistic regression analysis of Port reservoir height and revision risk (Exp(B) represents the odds ratio for revision compared to the reference height of 10.6 mm)

Port Reservoir Height (mm)	Exp(B)	95% CI (Lower-Upper)	*p*-value
10.6 (reference)	-	-	-
11.6	1.49	0.15–14.54	0.734
12.2	4.47	1.23–16.26	0.023
13.2	10.39	2.93–39.89	< 0.001

### Impact of Anti-VEGF therapy on revision risk

Among 662 patients, 63 (9.5%) received anti-VEGF therapy as part of their oncologic regimen. The incidence of port revision was significantly higher in this subgroup compared with patients not receiving anti-VEGF agents (*p* < 0.05). In multivariate logistic regression analysis, anti-VEGF therapy remained an independent predictor of revision, even after adjustment for port reservoir height and patient-related variables (Table 7). No similar association was observed with anti-EGFR or other targeted agents.

**Table 7:** Multivariate logistic regression analysis of Port reservoir height and Anti-VEGF therapy in relation to revision risk

Variables	Exp(B) (OR)	95% CI (Lower–Upper)	*p*-value
Anti-VEGF therapy (yes v. no)	2.934	1.17–7.36	0.022
Port reservoir height (mm)			
10,6 (reference)	-	-	-
11,6	1.574	0.16–15.47	0.697
12,2	4.409	1.21–16.06	0.024
13,2	9.998	2.81–35.62	< 0.001

Patients who received anti-VEGF therapy and were implanted with thicker ports (≥ 12 mm) represented the subgroup with the highest observed revision frequency. Anti-VEGF therapy was associated with a nearly three-fold increase in revision risk, while the thickest ports (13.2 mm) were linked to an approximately ten-fold higher risk compared with the reference group. Although the logistic model did not include an interaction term, the concurrent presence of both risk factors appeared to further amplify revision risk, highlighting the additive impact of treatment- and device-related variables.

### Patient-Related factors and comorbidities

Patient-related variables including age, sex, body mass index (BMI), smoking status, diabetes mellitus, hypertension, thyroid dysfunction, chronic corticosteroid use, and organ failure (renal, hepatic, or cardiac) were analyzed in relation to revision risk. None of these parameters showed a statistically significant association with the occurrence of port revision (all *p* > 0.05), as summarized in Table 8.

**Table 8:** Association between Patient-Related variables and revision requirement

Variable	*p*-value
Smoking	0.976
Diabetes mellitus	0.411
Hypertension	0.971
Thyroid dysfunction	0.142
Organ failure	0.710
Chronic corticosteroid use	0.653
No comorbidity	0.622

### Structural and material characteristics of the Port systems

The four port models differed in several physical and material parameters, including reservoir height, septum diameter, internal and external volume, overall device dimensions, and chamber composition (Table 9).

**Table 9:** Structural and material characteristics of the Port systems

Parameter	Brand A	Brand B	Brand C	Brand D	*p*-value
**Reservoir height (mm)**	10.6	11.6	12.2	13.2	< 0.001
**Internal volume (mL)**	0.36	0.60	0.24	0.50	0.37
**Weight (g)**	5.9	5.2	7.6	9.0	< 0.001
**Septum diameter (mm)**	13.0	13.0	12.1	12.0	< 0.001
**Overall dimensions (mm)**	13.5 × 13.5 × 10.6	14.5 × 14.5 × 11.6	30.8 × 22.6 × 12.2	17.5 × 13.5 × 13.2	< 0.001
**External volume (mL)**	6.1	7.7	8.5	9.8	< 0.001
**Chamber material**	Titanium + Polysulfone	Titanium	Titanium + Polyoxymethylene	Titanium + Polysulfone	0.21
**Septum material**	Silicone	Silicone	Silicone	Silicone	-

Statistical analysis revealed significant associations between revision risk and several structural variables: reservoir height (*p* < 0.001), septum diameter (*p* < 0.001), external volume (*p* < 0.001), overall dimensions (*p* < 0.001), and device weight (*p* < 0.001). In contrast, internal reservoir volume (*p* = 0.37) and chamber material (*p* = 0.21) showed no significant relationship with revision occurrence. Among all measured parameters, reservoir height and device weight demonstrated the strongest association with revision risk (both *p* < 0.001).

### ROC curve analysis

**Fig. 3:**
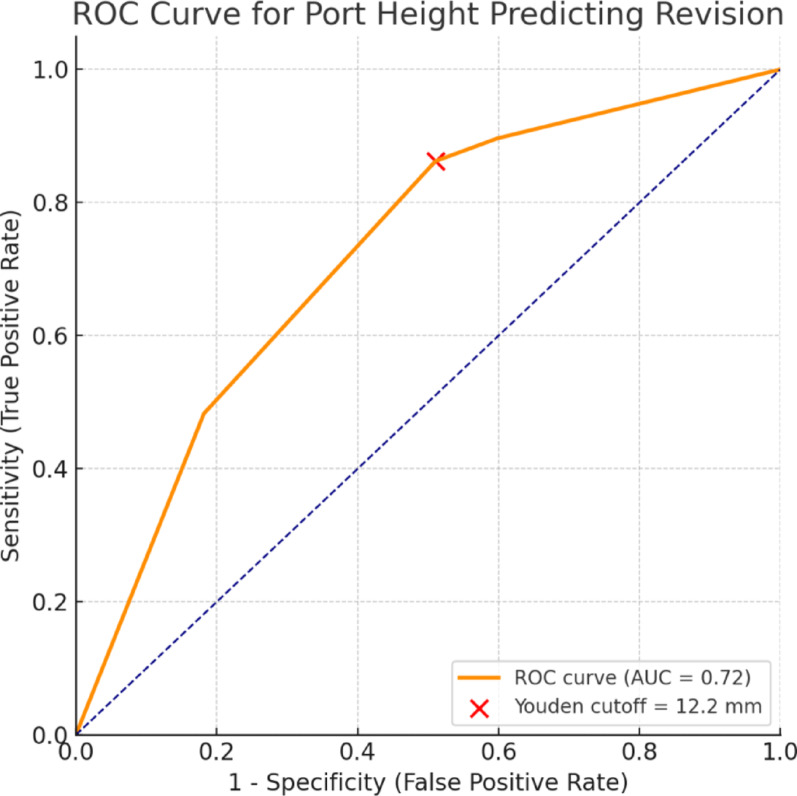
ROC curve demonstrating the predictive performance of port reservoir height for revision. The area under the curve (AUC) was 0.72, indicating good discriminatory ability. According to the Youden index, the optimal cut-off value was 12.2 mm (sensitivity = 0.86, specificity = 0.49). The red dot denotes the optimal threshold point. This threshold value (12.2 mm) corresponds exactly to the point where logistic regression analysis also showed a sharp increase in revision risk, reinforcing the clinical validity of the model

ROC curve analysis demonstrated a significant association between port reservoir height and the risk of revision due to skin erosion. The area under the curve (AUC) was 0.72, indicating acceptable discriminatory performance. The optimal threshold determined by the Youden index was 12.2 mm, yielding a sensitivity of 86.2% and a specificity of 48.9%. Ports with a reservoir height above this threshold were associated with a markedly higher incidence of revision. These findings suggest that a port reservoir height exceeding approximately 12 mm may represent a critical limit beyond which the risk of skin erosion increases substantially (Fig. 3).

### Statistical overview

Overall, statistical analyses demonstrated that both device-related and treatment-related variables played a decisive role in determining revision outcomes. Among all evaluated parameters, port reservoir height and overall device weight were the most powerful independent predictors, showing a consistent positive association with the likelihood of revision. Anti-VEGF therapy also remained a significant independent factor, reinforcing the influence of systemic treatment on local wound healing. In contrast, patient-related characteristics such as age, sex, body mass index, comorbidities, and chronic corticosteroid use showed no statistically meaningful relationship with revision risk (all *p* > 0.05). The findings suggest that mechanical stress, rather than patient physiology, may be the dominant determinant of skin integrity over implanted ports.

## Discussion

This study demonstrated that port reservoir height is an independent predictor of skin erosion requiring revision. In addition to reservoir height, several structural parameters, including device weight, external volume, and septum diameter, showed significant associations with revision risk, whereas internal volume and material composition did not. Together, these findings highlight that the overall structural profile of the port system plays a decisive role in the development of skin erosion.

The mechanical explanation for this association likely lies in the cumulative cutaneous pressure exerted by thicker and heavier reservoirs. Increased vertical height and surface bulk may compromise dermal perfusion, leading to localized ischemia and thinning of the overlying skin. This mechanism is consistent with previous reports suggesting that high-profile reservoir geometry predisposes to port exposure and erosion. Our findings extend these observations by identifying a measurable geometric threshold (approximately 12 mm) beyond which the risk of erosion rises sharply, as confirmed by both logistic regression and ROC analysis.

In this cohort, structural parameters such as reservoir height, external volume, and device weight appeared to act in concert. This suggests that these variables may represent different facets of a single mechanical phenomenon: the “overall bulk effect.” A bulkier device increases tension at the dermo-subcutaneous interface, leading to greater local pressure and reduced tissue perfusion over time. Although subcutaneous thickness was not routinely measured in this retrospective analysis, the consistent correlation across multiple mechanical parameters supports a geometric–pressure relationship that warrants further biomechanical investigation.

Anti-VEGF therapy was identified as another independent risk factor for revision. This association is biologically plausible given the role of VEGF in angiogenesis and wound healing. Inhibition of VEGF impairs capillary formation and delays tissue regeneration, thereby increasing susceptibility to wound dehiscence and erosion. Earlier studies, such as that by Erinjeri et al., have emphasized the timing of bevacizumab administration relative to port implantation, showing that shorter intervals significantly elevate the risk of wound complications [18]. Our data extends these findings by demonstrating that anti-VEGF therapy independently increases erosion risk, irrespective of port geometry. Patients with both a thick reservoir (≥ 12 mm) and anti-VEGF exposure constituted the subgroup with the highest observed revision frequency, suggesting a potential additive effect between systemic and mechanical risk factors.

Consistent with previous reports, none of the patient-related variables, including age, sex, body mass index, diabetes mellitus, hypertension, smoking, chronic corticosteroid use, thyroid dysfunction, or organ failure (renal, hepatic, or cardiac), showed a significant association with revision. This lack of correlation underscores that the principal drivers of skin erosion in this cohort were structural and pharmacologic rather than demographic or metabolic. Okazaki et al. similarly reported that age, sex, hypertension, diabetes, and BMI were not significantly associated with long-term port complications in a 150-case gastric cancer series, except for a mild BMI-related trend in catheter thrombosis rather than erosion [19]. Our findings are consistent with theirs for demographic and metabolic factors, while additionally demonstrating that smoking, steroid therapy, thyroid dysfunction, and major organ failure also lacked significant influence on port erosion risk. From a clinical standpoint, this implies that even in well-controlled patients without major comorbidities, mechanical load and impaired wound angiogenesis remain critical determinants of local complications.

Previous literature on port-related complications has largely focused on infection, thrombosis, or dehiscence, whereas few studies have examined the geometric or structural characteristics of port design. Our findings complement earlier reports by Burris and Weis, who noted that high-profile ports were more prone to erosion and recommended selecting lower-profile devices to minimize local pressure [13]. Beyond geometric configuration, material-related aspects have also been explored. Guiffant et al. demonstrated that repeated needle punctures and surface irregularities in plastic-based ports can alter local flow patterns and wall shear stress, potentially leading to mechanical degradation over time [6]. In our series, however, material composition itself was not a significant determinant of erosion risk, suggesting that macroscopic design parameters, such as reservoir height, external volume, and weight, play a more dominant role than material type. The current study therefore integrates these biomechanical considerations into clinical evidence, providing quantitative support for the role of port geometry in complication risk.

Burris and Weis emphasized that in thin or cachectic patients, high-profile ports increase subcutaneous pressure and should therefore be avoided in favor of lower-profile devices [13]. Zawacki et al., who analyzed 195 ports implanted in 189 patients, similarly reported that high-profile ports carried a significantly greater risk of skin erosion [20]. Both studies also noted that Bevacizumab therapy may delay wound healing, a finding consistent with our own observations regarding anti-VEGF agents. However, the concept proposed by Burris and Zawacki that cachexia and low body mass index are independent risk factors for erosion was not confirmed in our series. The absence of a significant correlation between BMI and erosion may reflect the relative homogeneity of our cohort and the standardized implantation technique performed by a single surgeon. These findings suggest that mechanical factors, such as port height, external volume, and weight, play a more dominant role than individual body habitus in determining the risk of skin erosion.

## Conclusions

These findings emphasize the importance of device geometry in long-term port safety and suggest that selecting lower-profile ports, particularly in patients receiving anti-VEGF therapy, may help minimize local complications. In practical terms, thinner and lighter-profile ports should be favored over bulkier devices, and when anti-VEGF therapy is planned, choosing the slimmest possible port design becomes a critical safety consideration. Further prospective and biomechanical studies are warranted to refine design parameters and validate these results across diverse clinical settings.

Finally, this study underscores the importance of interdisciplinary collaboration between clinicians and biomedical engineers. Future investigations integrating finite element modeling, pressure mapping, and real-world mechanical testing could refine our understanding of the mechanical-biological interface underlying port erosion. Establishing standardized port dimensions and design criteria may ultimately contribute to the development of safer, evidence-based vascular access systems for oncologic care.

## Limitations

This study has several limitations. First, its retrospective design introduces the possibility of selection and documentation bias inherent to chart-based analyses. Second, all procedures were performed at a single center by the same surgeon, which enhances procedural consistency but may limit the generalizability of the findings. Third, subcutaneous tissue thickness was not measured, preventing assessment of its potential contribution to skin erosion. Fourth, all port placements were performed using the anatomical landmark technique without ultrasound guidance, reflecting the routine practice in a low-resource institutional setting. Finally, while this study statistically identified a 12-mm height threshold for increased revision risk, the lack of prospective validation and biomechanical modeling limits the strength of this conclusion and warrants further controlled investigations to confirm this geometric relationship.

## Data Availability

The datasets generated and analyzed during the current study are available from the corresponding author upon reasonable request.
